# Highly sensitive *in vitro* bioassay for luteinizing hormone and chorionic gonadotropin allowing their measurement in plasma

**DOI:** 10.1530/RAF-21-0045

**Published:** 2021-11-11

**Authors:** Danièle Klett, Yves Combarnous

**Affiliations:** 1 Institut National de la Recherche Agronomique et environnementale (INRAe), Centre National de la Recherche Scientifique (CNRS), Université de Tours, Joint Laboratory of Reproductive Physiology and Behaviors (PRC), Nouzilly, France

**Keywords:** Leydig cell, cyclic AMP, luciferase, forskolin, oxytocin

## Abstract

In previous studies, we had shown the synergistic effect of 10^−5^ M forskolin (FSK) on the detection threshold of the cyclic AMP response to luteinizing hormones (LH) and chorionic gonadotropins (CG) from various species in the mouse Leydig tumor cell (mLTC) cell line. Independently, we started to study the effect of 10^−12^–10^−6^ M oxytocin (OXT) also on the cyclic AMP response to LH and CG preparations on these same cells and found an amplifying effect on the luminescence response caused by gonadotropins. The aim was then to explore the effects of 10^−12^–10^−6^ M OXT on the gonadotropin-induced cAMP response, in the presence or absence of 10 µM FSK to optimize the assay down to a sensitivity compatible with the detection of the circulating concentrations of these hormones in various species. Finally, the optimization relies on three independent phenomena: (1) the inhibition of nucleotide phosphodiesterase by IBMX (3-isobutyl-1-methylxanthine) to avoid cAMP degradation; (2) the strong synergy of 10 µM forskolin with low concentrations of LH or CG during the 1-h luminescence measurement; (3) the stimulatory effect of 10^−8^M OXT on the amplitude of transfected cAMP-sensitive luciferase response. By doing this, the detectable concentrations are at the 1–10 pg/well (pM range) for the LHs and CGs from various species. The bioactivities of circulating LHs and CGs in blood or urine are therefore expected to be measurable in 10 µL-plasma samples from mammalian species and maybe others. Indeed, a preliminary study with equine and donkey plasma samples shows that the measured bioactivity was fully inhibited by a specific MAB against the receptor-binding region of equine LH (eLH) and equine CG (eCG), thus eliminating a possible response due to interfering substances other than eLH or eCG. From these data, it is expected that the bioactivity profiles of these hormones will be measurable in the blood of human, equine, and ovine species and very likely in rodents, ruminants, and hopefully in most other mammalian species.

**Lay summary:**

Luteinizing hormone (LH) plays a central role in controlling ovary and testicle functions in many animals, including humans. The highly sensitive method, known as an assay, described in this paper, measures the biological activity of LH in the blood of mammals. The assay is performed in culture of cells derived from mouse testicles in the presence of factors that diminish the detection threshold for LH. The knowledge of the bioactive LH concentration dynamics in the blood is very informative about the reproductive status of male and female mammals. This new* in vitro* bioassay provides a powerful tool to get this information.

## Introduction

The mouse Leydig tumor cell line (mLTC) (Rebois 1982) has been used for years to study the signaling pathway downstream of the binding of LHs and CGs to their LH receptor named LHR in almost all vertebrate species and LHCGR in the primates and equids, who are the only groups having chorionic gonadotropin (CG) (Rebois & Fishman 1984, Kellokumpu 1987, El-Hefnawy *et al.* 2000, Wang *et al.* 2007, Mendoza-Villarroel *et al.* 2014). Since the mouse LHR can bind to LH and CG from numerous species, we and others have used this cell line to study the response to various LH and CG preparations at the levels of intracellular cyclic AMP accumulation (Dufau *et al.* 1995, Nikula *et al.* 1999, Evaul & Hammes 2008, Klett *et al.* 2016, Nguyen *et al.* 2018, 2019). For this purpose, we transfected the cells with an expression vector encoding a cAMP-dependent luciferase (Glosensor) and measured the luminescence of oxiluciferin produced as a response to cAMP accumulation.

An outstanding property of mLTCs is that their adenylate cyclase(s) is/are insensitive to treatment with forskolin (FSK) alone (Klett *et al.* 2016). Nevertheless, FSK exhibits a strong synergy with very low concentrations of LH or CG that do not show any stimulating activity in its absence to reach a much lower threshold for LH and CG detection (Nguyen *et al.* 2018).

Besides, it has been known for a long time that Leydig cells possess oxytocin receptors (OXTR) (Frayne & Nicholson 1995, Einspanier & Ivell 1997). We have thus explored the effects of an extensive range of oxytocin (OXT) concentrations on the cAMP response of mLTCs and found that it strongly diminished the detection limit for gonadotropins down to a threshold compatible with their measurement in biological fluids.

## Materials and methods

mLTC (Rebois 1982) were obtained from the American Tissue and Cell Collection (LGC Standards, Molsheim, France). For the assays, the cells were thawed, expanded, and used from passes P6–P20. About 80,000 cells were seeded per well on a 96-well Greiner white/clear bottom plate (Dutscher, Bernolsheim, France) and incubated at 37°C under 5% CO_2_ in 200 µL-supplemented RPMI growth medium. The hormone preparations used in the present study were human chorionic gonadotrophin (hCG) from Hepartex (Saint-Cloud, France) with an activity of 7482 IU/mg, recombinant human LH (hLH) from Sigma-Aldrich with an activity of 4500 IU/mg, pituitary hLH (SIAFP-4261A), as well as highly purified equine LH (eLH CY937), equine CG (eCG NZY02), ovine LH (oLH CY1072), and bovine LH (bLH CY1280) from our laboratory. FSK and OXT were purchased from Sigma.

The assay presented in this paper is based on our previously described methodology (Klett *et al.* 2016).

In brief, the mLTC cells, at 80% confluency, were transiently transfected with pGlosensor^TM^ 22F cAMP plasmid (Promega) using XtremeGENE HP DNA transfection reagent (Roche). DNA (100 ng per well) and transfection reagent (0.3 µL per well) were first mixed together and incubated in serum-free RPMI medium 30 min before transfection. Supernatants were aspirated and replaced with 100 µL of supplemented RPMI medium containing 10% transfection mix. After 24 h at 37°C under 5% CO_2_, supernatants were replaced with 100 µL of an equilibrium medium, composed of serum-free RPMI medium containing 10^−3^ M IBMX and 4% (v/v) GloSensor™ cAMP Reagent. The plate was then incubated for 2 h at room temperature. The molecules to be tested were added over all or part of this incubation period when required. Stimulation of the cells began immediately before luminescence recording, by adding 11 µL per well of the 10×-concentrated doses of hormones and/or of FSK. The Graph-Pad Prism 5.01 package was used for the determinations of the area under curve of luminescence kinetics and the statistical comparisons of data (mean and errors on triplicate luminescence at each recording time, repeated ANOVA to compare conditions). At least three independent experiments were performed for each condition, each with triplicate kinetics. Representative experiments are shown in the results section and figures.

We previously reported that 10 µM FSK exerted a strong synergy with the cAMP response of mLTCs to LH and CG preparations (Klett *et al.* 2016, Nguyen *et al.* 2018). To further reduce the detection threshold of this assay, we explored the effects of preincubating the cells for 0–4 h with OXT concentrations ranging from 10^−12^ to 10^−6^ M on the cAMP response to gonadotropins in the presence or absence of FSK. The data showed that OXT indeed considerably increases the cAMP response amplitude in mLTC cells and together with IBMX and FSK allows the determination of bioactive LH and CG concentrations in blood.

## Results

### Effect of 10^−12^–10^−6^M OXT on the cAMP-dependent luciferase activity response to LH and CG preparations

**Figure 1:**
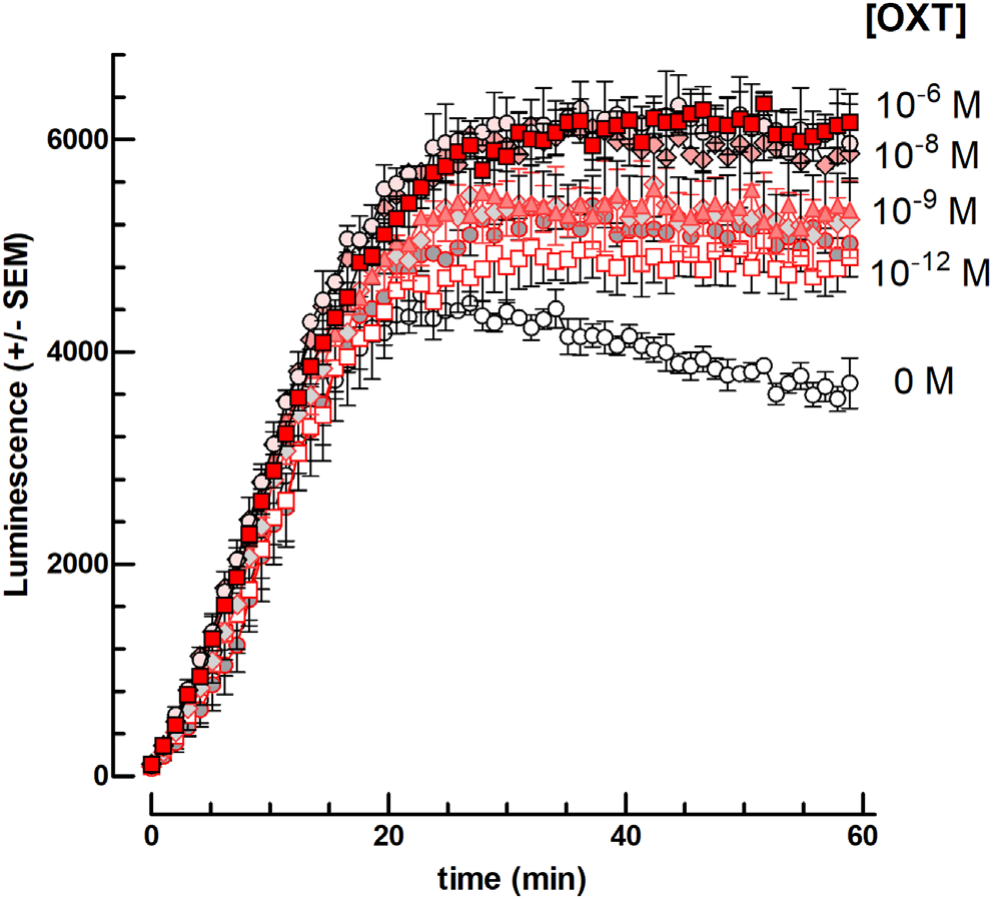
Effect of 10^−12^–10^−6^ M OXT on the cAMP-dependent luciferase response to 10 ng hCG per well in mLTC cells. The cells were preincubated for 1 h in the absence (0 M) or presence of 10^−12^, 10^−11^, 10^−10^, 10^−9^, 10^−8^, 10^−7^, or 10^−6^ M OXT (*n* = 3) before the addition of 10 ng hCG per well (2.9 nM final) and initiation of luminescence recording for 1 h.

In the first set of experiments, we examined the effects of OXT added to the cell medium simultaneously with gonadotropins in the presence of 1 mM IBMX, without FSK. Figure 1 shows that OXT provokes a dose-dependent enhancement of the intracellular cAMP accumulation under hCG stimulation.

### Time- and dose-dependence of the effect of OXT on the cAMP-dependent luciferase activity

**Figure 2:**
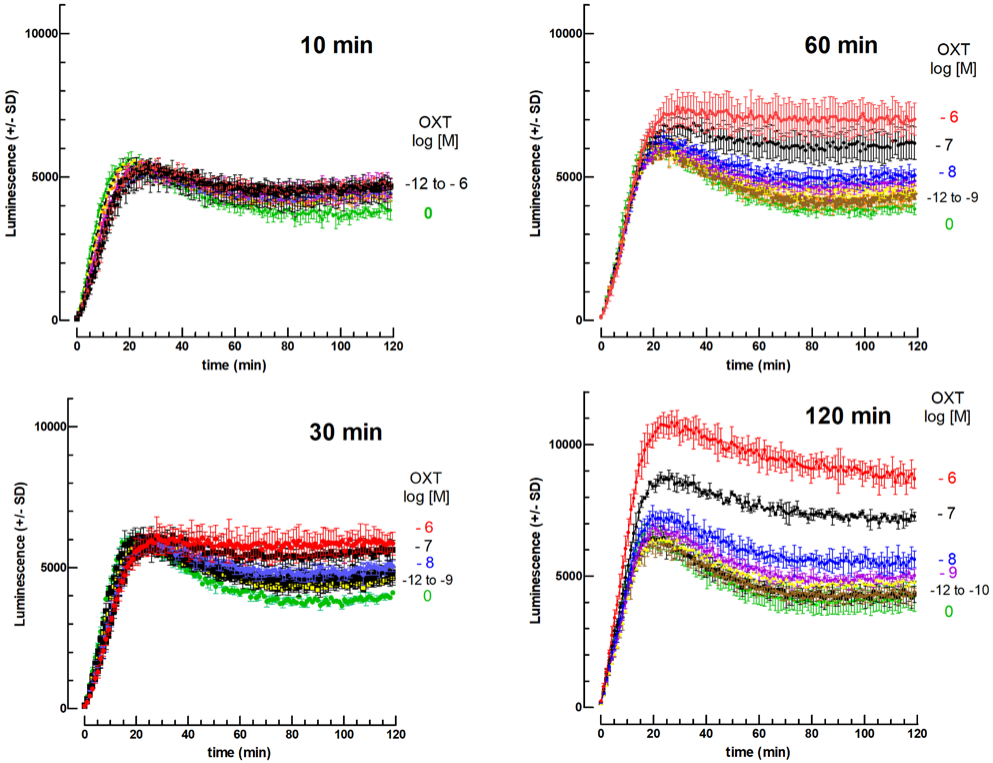
Effect of 10^−12^–10^−6^ M OXT added between 10 min and 2 h before 10 ng hCG (2.9 nM) on the cAMP-dependent luciferase response of mLTC cells. The cells were preincubated for 10, 30, 60, or 120 min in the absence (0) or presence of 10^−12^, 10^−11^, 10^−10^, 10^−9^, 10^−^^8^, 10^−^^7^, or 10^−^^6^ M OXT (*n* = 3) before the addition of 10 ng hCG per well (2.9 nM final) and initiation of luminescence recording for 2 h. This a representative experiment out of four.

When OXT is preincubated with the cells before adding gonadotropins, a dose- and time-dependent increase in the cAMP response is observed (Fig. 2) in the absence of FSK. The increasing effect of OXT preincubation on the cAMP response of mLTCs to hCG was also observed with the other gonadotropins tested (hLH, oLH, eLH, and bLH).

### Effect of 2-h preincubation with 10^−12^–10^−6^M OXT on the cAMP-dependent luciferase response to hCG in the presence of FSK

**Figure 3:**
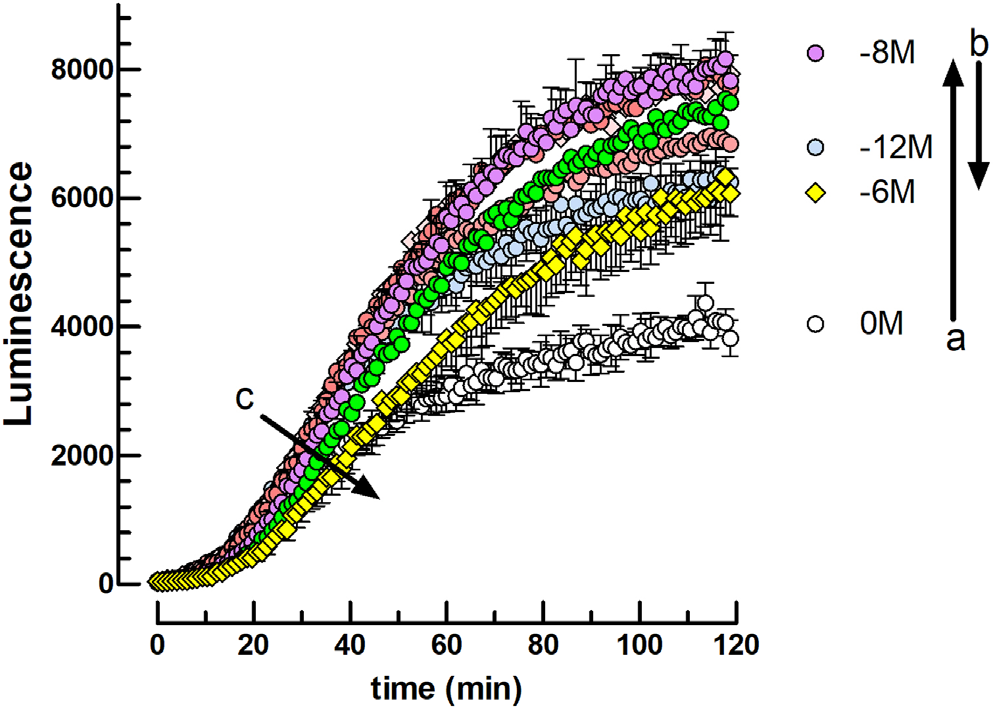
Effect of 10^−12^–10^−6^ M OXT 2-h preincubation on the cAMP-dependent luciferase response to FSK + hCG in mLTC cells. The cells were preincubated for 120 min in the absence (0) or presence of 10^−12^, 10^−11^, 10^−10^, 10^−9^, 10^−^^8,^ 10^−^^7^, or 10^−^^6^ M OXT (*n* = 3) before the addition of FSK (10 μM final) and 312 ng hCG per well (90 pM final) immediately followed by initiation of luminescence recording for 2 h. Arrows: (A) indicates the response increase between 0 and 10^−8^ M OXT and (B) its decrease between 10^−8^ and 10^−^^6^ M OXT after 120 min. (C) Indicates the response rate decrease between 10^−^^12^ M and 10^−^^6^ M OXT at 40-min incubation time.

In a second set of experiments, we explored the dose-dependent effect of OXT after a 2-h preincubation of the cells before stimulation by hCG together with FSK. The data in Fig. 3 show the kinetics of intracellular cAMP accumulation response to 0.312 ng hCG/well (90 pM). At this low hCG concentration, the positive effect exerted by OXT on response amplitude is partially counterbalanced by a negative effect of the higher OXT concentrations on the response rate. Consequently, an optimal 10^−8^M OXT concentration is observed in these experimental conditions.

### Effect of 10^−12^–10^−6^M OXT on the cAMP response to various LH and CG preparations in the presence of FSK

In the third set of experiments, we explored whether the other human (hLH) and animal gonadotropins (eLH, oLH, bLH, and eCG) in the presence of 10 µM FSK, also exhibited enhanced cAMP responses after a 2-h preincubation of mLTC cells with 10^−6^M OXT.

**Figure 4:**
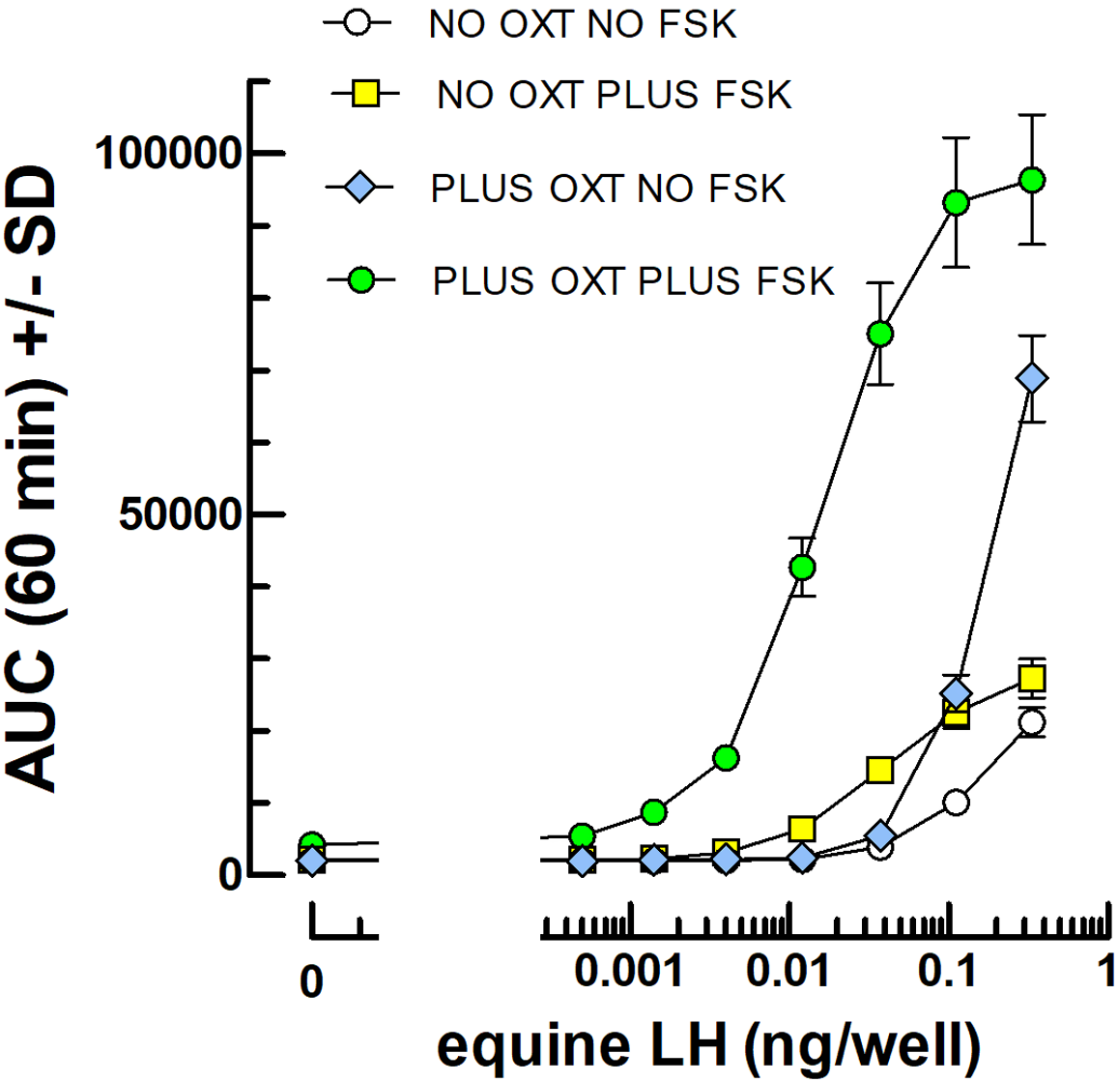
cAMP-dependent luciferase dose–response curves for equine LH in the absence or presence of FSK and OXT, alone or together. The cells were preincubated for 2 h in the absence or presence of 10^8^M OXT (*n* = 3) before the addition of FSK (0 or 10 μM final) and 0–0.37 ng eLH per well immediately before the start of luminescence recording for 1 h. The AUC of triplicate kinetics for each condition were calculated and are shown (mean ± s.d.) in the figure.

**Figure 5:**
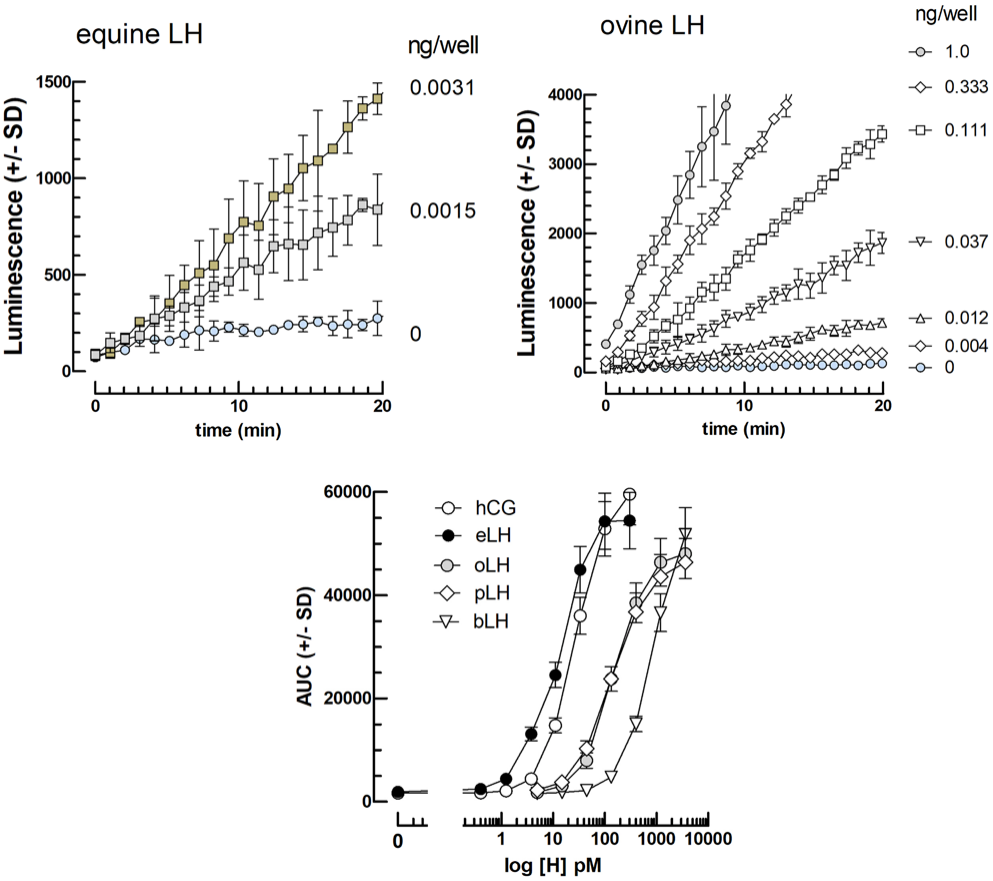
Upper panel: Kinetics of cAMP-dependent luciferase responses to equine LH and ovine LH. The mLTC cells were preincubated for 2 h in presence of 108 M OXT (*n* = 3) before the addition of FSK (10 μM final) and 0–0.025 ng eLH per well (left) or 0–1.0 ng oLH per well (right) immediately before the start of luminescence recording for 20 min. Lower panel: Dose–response curves for equine, ovine, porcine and bovine LHs, and human CG. The AUC of triplicate kinetics for each condition in the upper panel for equine (black symbols) and ovine LH ( gray symbols) were calculated and are shown (mean ± s.d.) in the figure as a function of each hormone molar concentration. The AUC values for the other hormones (hCG, pLH, and bLH ; open symbols) were calculated from kinetics (not shown) obtained the same way.

Figure 4 shows that OXT alone exerts a strong synergistic effect on the luminescence response to equine LH, but at the same time reduces sensitivity by raising the detection threshold. This latter unfavorable effect of OXT is overcome by the previously described favorable effect of FSK on the LH detection threshold in this system. That elicited a significantly greater response using a 2-h preincubation of mLTCs with 10^−8^M OXT followed by a 1-h incubation of gonadotropin preparations under study in the presence of 10^−5^M FSK (Fig. 5).

We then looked for the lowest concentrations of these different hormones that could be significantly detected using a 2-h preincubation of mLTCs with 10^−8^M OXT and 10^−5^M FSK during the 1-h stimulation by the various gonadotropins. The detection thresholds were calculated on the minimum concentration giving a response two s.d. above the corresponding control. These thresholds were 1.4 pg/110 µL-well for hLH (i.e. 0.45 pM), hCG (0.40 pM), and eLH (0.40 pM), 4.0 pg/110 µL-well for oLH (1.29 pM), 12 pg/110 µL-well for eCG (2.47 pM), and 37 pg/well for bLH (12.0 pM).

### Mechanism of OXT action on the cAMP-dependent luciferase response to LHs and CGs

In a preliminary experiment, 2-h preincubation with OXT in the presence of cycloheximide led to a quick and robust inhibition of the subsequent stimulation of the cAMP-dependent luciferase stimulation by hCG or LH.

In order to test whether the OXT effect could be due to stimulation of the translation of the Glosensor luciferase plasmid, we measured the luciferase response to db-cAMP (500 µM) or eLH (0.3 nM) after a 2-h preincubation of mLTC cells without or with 10^−^^12^–10^−6^ M OXT.

**Figure 6:**
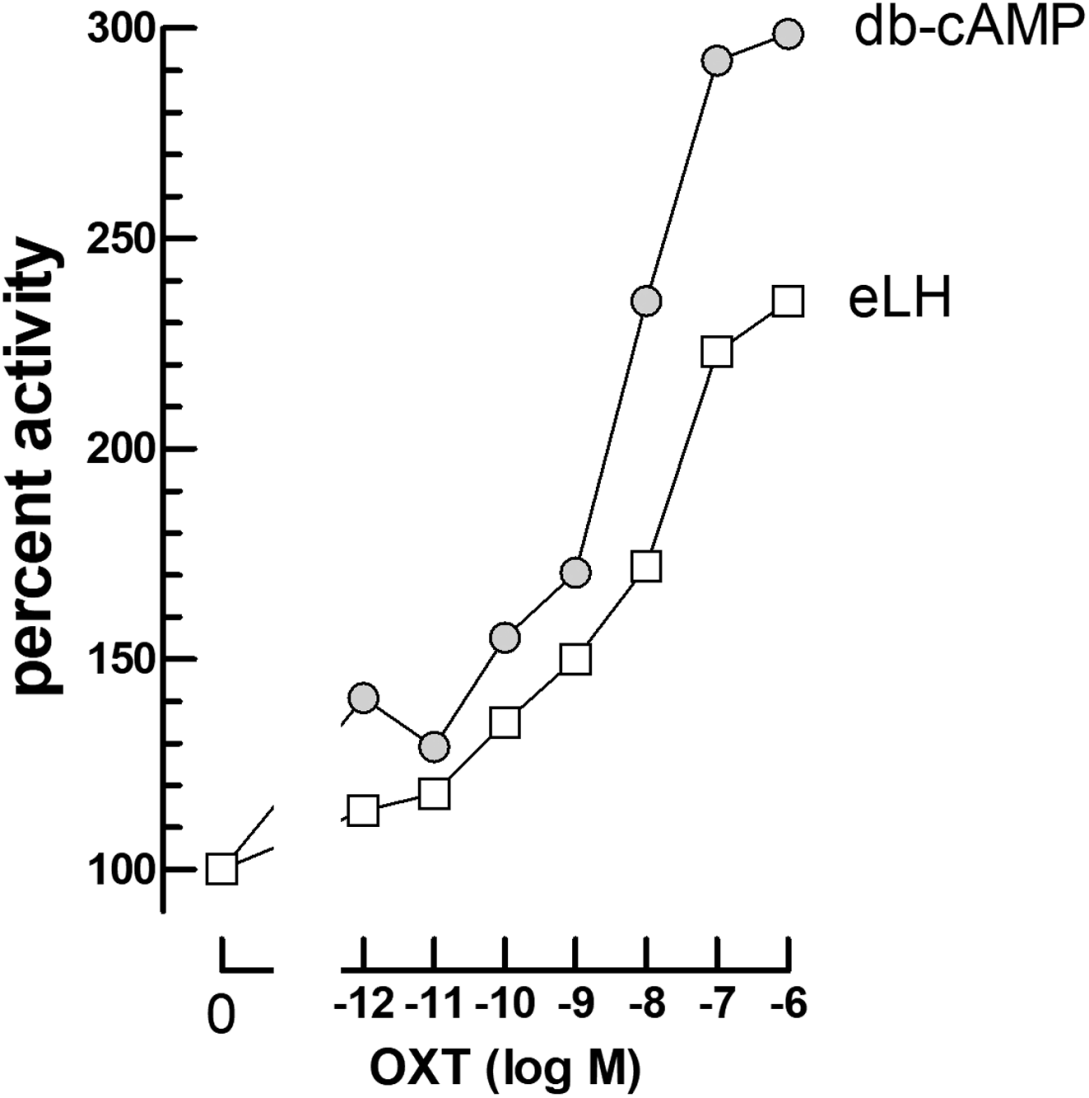
Effect of 10^−12^–10^−6^ M OXT added 2 h before db-cAMP or equine LH on the cAMP-dependent luciferase response in mLTC cells. The cells were preincubated for 120 min in the absence (0) or presence of 10^−12^, 10^−11^, 10^−10^, 10^−9^, 10^−^^8^, 10^−^^7^, or 10^−^^6^ M OXT (*n* = 3) before the addition of FSK (10 μM final) and either db-cAMP (500 μM final) or eLH (300 pM final) immediately followed by initiation of luminescence recording for 2 h. The initial 100% values correspond to the respective responses to db-cAMP and equine LH in the absence of OXT. It must be stressed that in the absence of OXT, the AUC for 500 μM db-cAMP is less than 1% that for 300 pM equine LH.

The data in Fig. 6 show that the luminescence responses are similarly increased as a function of OXT concentration, under stimulation by db-cAMP or equine LH. It must be stressed however that the absolute response to 500 µM db-cAMP is less than one percent that to 300 pM eLH. In spite of this low effect by db-cAMP, there is good parallelism of its dose-dependent OXT potentiation with that of the potention of eLH response. This argues in favor of a stimulating effect of OXY on the cAMP-dependent luciferase concentration in the cells.

### Measurement of equine LH in plasma

**Figure 7:**
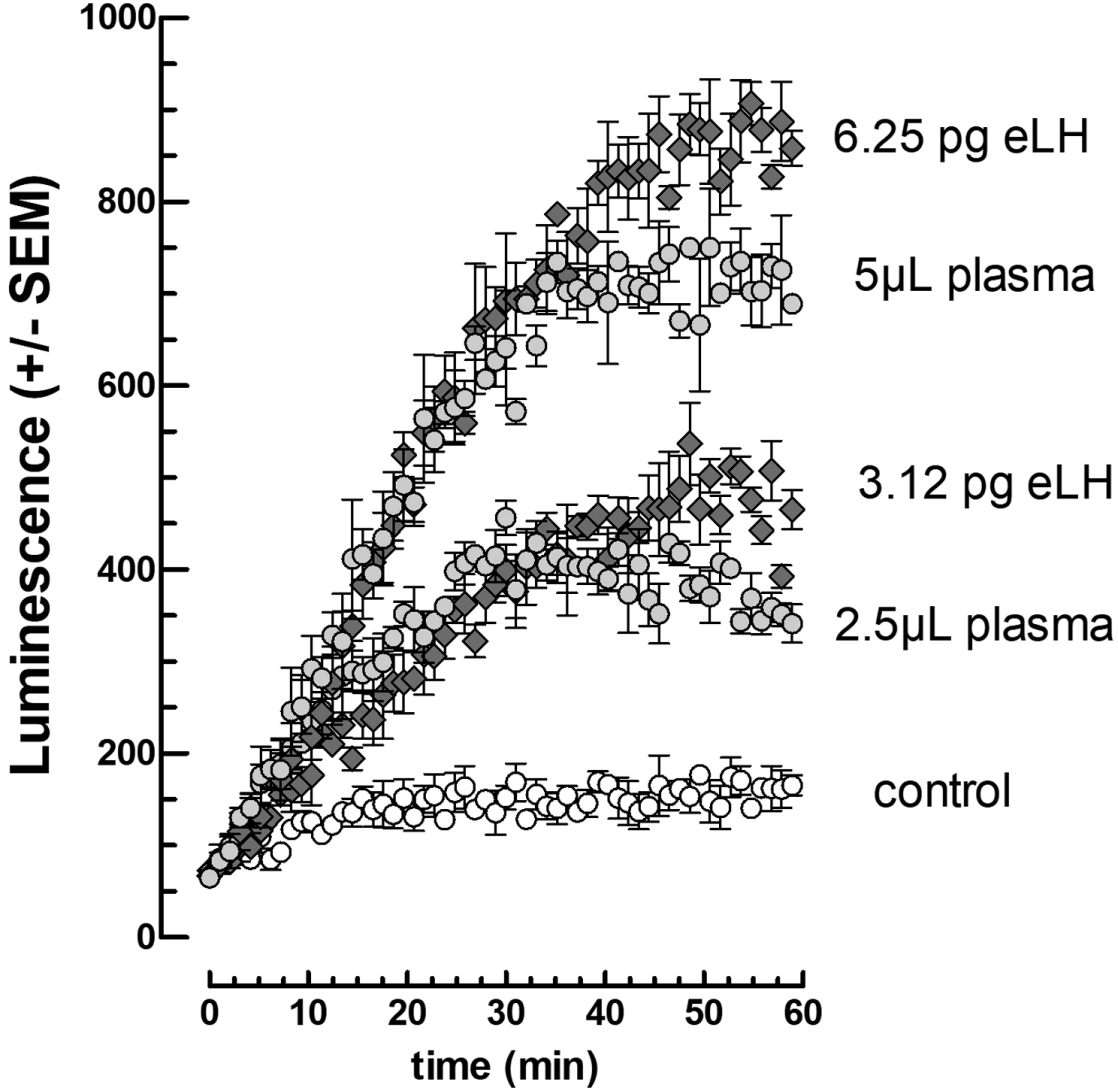
Stimulation kinetics of cAMP-dependent luciferase activity by equine LH and mare plasma. The mLTC cells were preincubated for 2 h in presence of 10^8^M OXT (*n* = 3) before the addition of FSK (10 μM final) and 0–6.25 pg equine LH or 0–5 μL mare plasma (non-breeding season) per well and immediate start of luminescence recording.

Since we use 11 µL plasma per well in a final volume of 110 µL, expected concentration thresholds in plasmas are of course 10-fold higher than those determined above in the wells, that is, 12 pg/mL for the equine LH and 12–336 pg/mL for the hormones from the other species studied so far. In agreement with these expectations, we could measure LH in mare plasma taken during the anovulatory season when the LH level is known to be at its lowest (Fig. 7). In agreement with these expectations, we could measure LH in these conditions.

### Authentication and measurement of LH in equine plasma and donkey serum

In this experiment, we measured the LH activity in plasma samples taken from mares during seasonal anestrus. To ascertain that the observed response is indeed due to LH only and not to some interfering substance(s), we studied the inhibition of this activity by a specific inhibitory MAB directed against epitope A in eCG and eLH. This epitope has been previously shown to belong to the binding region of eCG and eLH with their LH and FSH receptors (Chopineau *et al.* 1993).

**Figure 8:**
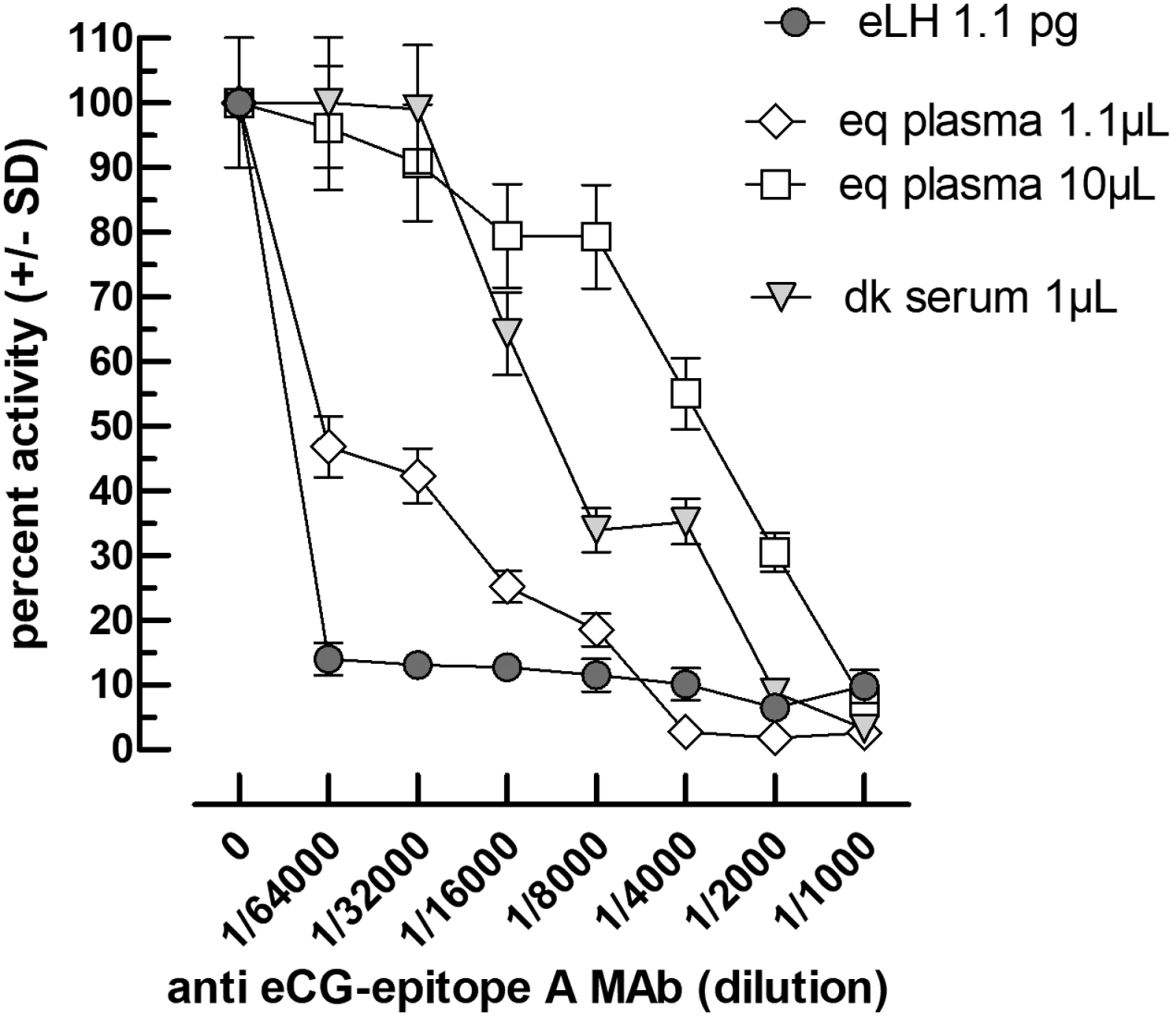
Inhibition of eLH and plasma activities by receptor-binding anti-eCG Mab. The equine LH (11 pg/mL) or mare plasma (1/10 or 1/100) or donkey (dk) serum (1/100) were mixed with anti-eCG MAB at final dilutions from 1/1000 to 1/64,000. The 100% value corresponds to the kinetics AUC of each material in the absence of the anti-eCG antibody.

The data in Fig. 8 show the activity of the mare plasma compared to highly purified eLH and demonstrate complete inhibition of its activity by the 89A2 anti-eLHCG epitope A MAB (Chopineau *et al.* 1993).

### Effect of luciferase plasmid concentration

**Figure 9:**
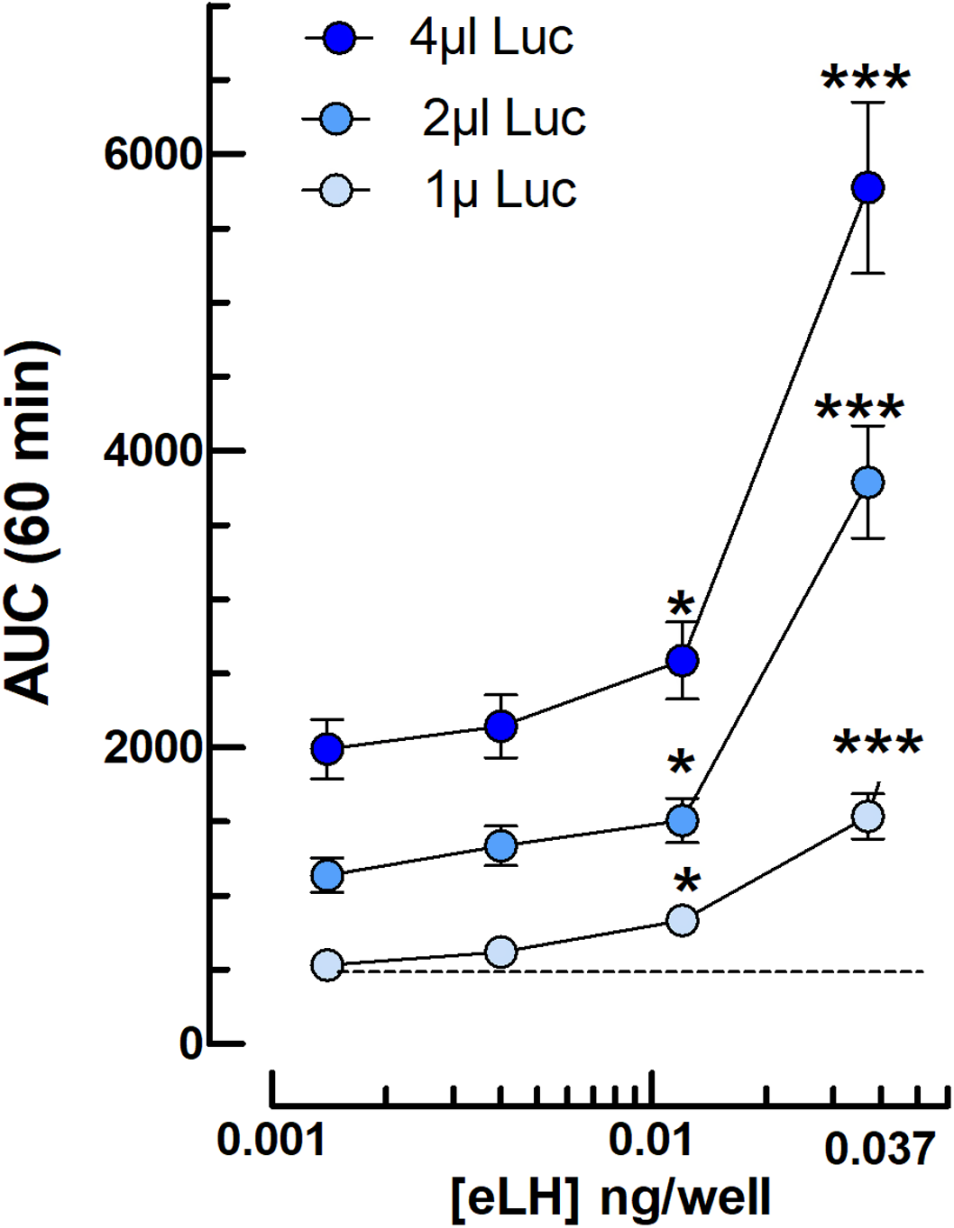
Effect of the Glosensor substrate concentration on the dose-dependent cAMP-dependent luciferase luminescence response to equine LH in mLTC cells. One, 2, or 4 μL of Glosensor substrate are diluted per 100 μL of serum-free culture medium containing 1 mM IBMX and 10^−8^ M OXT before addition to the cells for the 2-h preincubation period. The shown hormone concentrations are added after FSK (10 μM final), immediately before luminescence recording (*n* = 3). *P = 0.95 is the lowest value of eLH concentration outside of the control value ± 2 s.d. (0.011 ng/well) and the ***P = 0.95 detection threshold is the lowest value of eLH concentration outside of the control value ± 3 s.d. (i.e. 0.033 ng/well).

We have tested whether an increase in cAMP-dependent luciferase plasmid concentration during the transfection step affects the luminescence response to hormones. The data (Fig. 9) show that there is indeed a proportional dose-dependent decrease in the luminescence response with reduced concentrations of the transfected plasmid. Nevertheless, we found no change in the detection threshold of the assay. Thus, it is possible to adapt the plasmid concentration in this range to accommodate the sensitivity of the luminometer used and reduce the cost of the assay.

## Discussion

In previous papers, we have shown the favorable effect of FSK to significantly lower the detection threshold for LH and CG preparations in the cAMP assay making use of cAMP-dependent luciferase and Glosensor substrate in mLTC cells (Klett *et al.* 2016, Nguyen *et al.* 2018). In this article, we demonstrate that a 1–4-h preincubation of the cells with 10^−12^–10^−6^ M OXT before gonadotropin addition can increase the luminescent response amplitude thereby lowering the detection threshold for these gonadotropins. The 2–4-h preincubation of mLTCs with 10^−8^ M OXT plus the presence of 10^5^ M FSK together with LH or CG during the luminescence recording period permitted LH and CG bioactivity detection in the picomolar range. This range is compatible with the hormone concentrations in the blood of all the mammalian species tested so far and even much lower for some of them. Considering this low range, we will check in future studies whether the bioactivities of LH and CG can be measured in plasmas from various species under several physiological conditions. Urine samples will also be tested to avoid blood draws which can be stressful in humans and animals or impossible in many wild species.

Due to the conservation of functional structures in gonadotropins during evolution, cross bioactivities have been reported between gonadotropins from various vertebrate species tested in rodents. Thus, it will be determined whether bioactive LH concentrations can be measured in the blood of birds as well as of reptiles, amphibians, or fishes.

Such an assay will also allow to study the structural properties of LHs and CGs at physiological concentrations and to detect them in the blood of various species under varying physiological and physio-pathological conditions.

The data show that it is possible to detect the LH activity in an anovulatory non-pregnant Welsh mare. This activity can be abolished entirely with a low concentration of a specific Mab directed against the hormone domain interacting with its receptor. These data show that the stimulation observed in these conditions is only due to equine LH and not some other unknown factor.

The threshold for LH detection is in the same order of magnitude for oLH, hLH, and hCG (Fig. 5) and only slightly higher for bovine and porcine LH (not shown). It is thus hoped that this bioassay should be helpful in all wild and farm mammals, and maybe in other vertebrate species.

**Figure 10:**
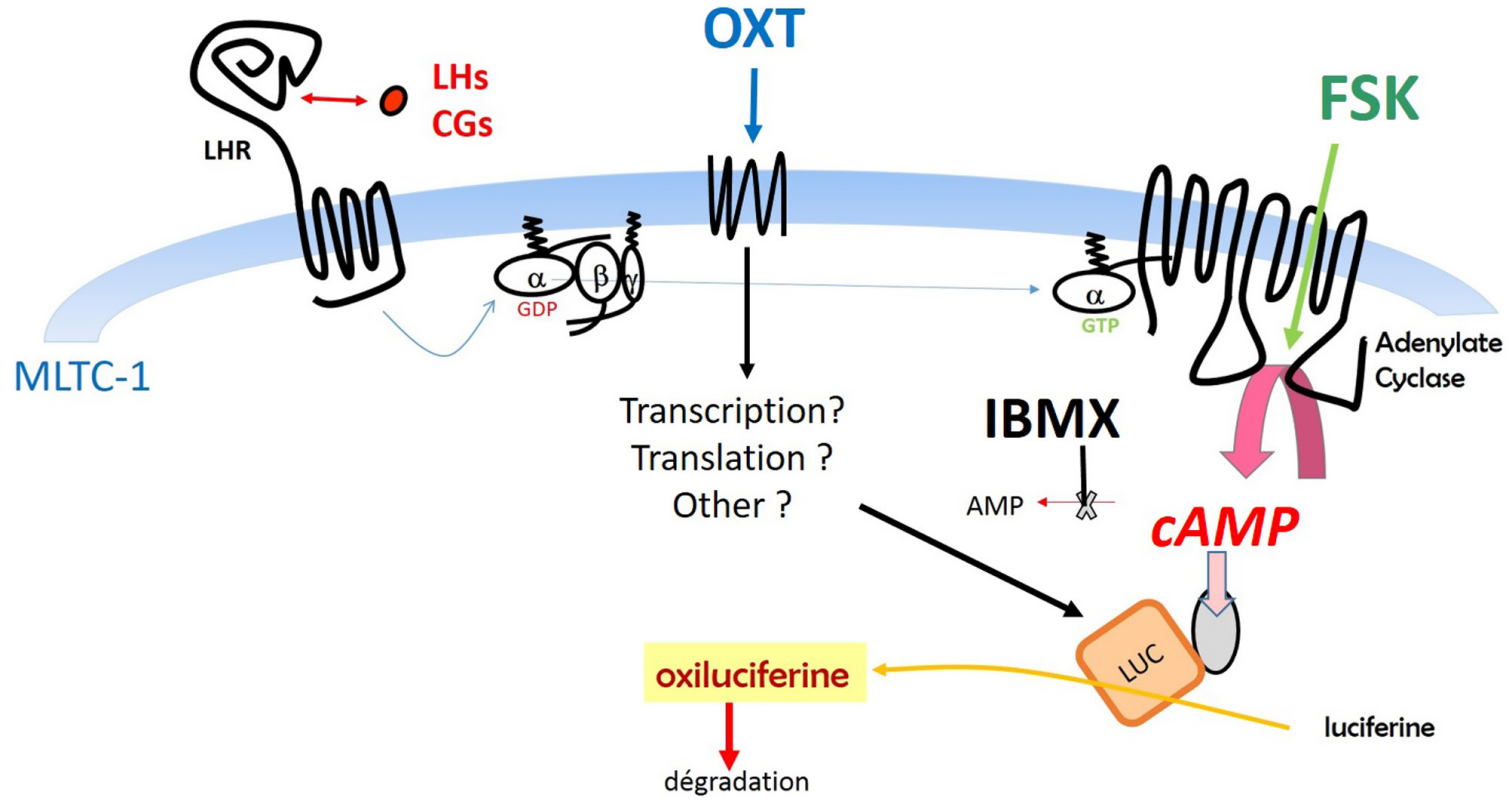
Graphical abstract showing the impact sites of FSK, IBMX, and OXT. Together they contribute to strongly lower the detection threshold as well as the amplitude of LH and CG-dependent luminescence response. IBMX by inhibiting cAMP hydrolysis by phosphodiesterases contributes to the enhancement of cAMP accumulation in mLTCs. FSK lowers the gonadotropin concentrations stimulating cAMP biosynthesis by adenylate cyclases. OXT acts through its receptors and requires protein translation and synthesis to provoke its large positive effect on the amplitude of the cAMP-dependent stimulation of luminescence. The hypothesis shown here is that this effect is due to an increase of the expression of the transfected cAMP-dependent luciferase is the more likely but does not exclude other possible mechanisms.

It is interesting to point out that the OXT effects in our experiments are abolished by cycloheximide thus indicating that at least one step in the observed potentiating effect by OXT is dependent on protein translation. The simplest and most straightforward hypothesis is that OXT stimulates the translation of the transfected cAMP-dependent luciferase. If this is true, there must also be a potentiating effect on the db-cAMP-dependent response through direct stimulation of the cAMP-dependent luciferase. It is indeed the case (Fig. 6) as 10^−8^ M OXT exerts a 2.5–3.0-fold-potentiating effect on the response to both eLH and db-cAMP. Nevertheless, it must be noticed that the absolute luminescence response to 500 µM db-cAMP is less than 1% that to 300 pM equine LH. The low response to db-cAMP might be due to low penetration in mLTC cells or due to low affinity toward the artificial cAMP-dependent luciferase. In spite of this low effect by db-cAMP, there is good parallelism of its potentiation by OXT with that of the potention of eLH response. This parallelism is in agreement with the view that the potentiating effect by OXT of the cAMP-dependent luminescence response to gonadotropins is due to the stimulation of the expression of the cAMP-dependent luciferase either at the level of transcription or translation (Fig. 10). Due to the rapidity of the potentiating effect (Fig. 2), it is more likely that OXT stimulates the protein synthesis step but its action at the transcriptional level cannot be excluded with certainty.

In the present study, we have used the mLTC Leydig cell line that we have been using for many years. Other teams have used the MA-10 Leydig cell line with great success for deciphering LH and CG mechanisms of action. The protocol we have developed and reported herein could probably be successfully applied to the MA10 cell line as well.

## Declaration of interest

The authors declare that there is no conflict of interest that could be perceived as prejudicing the impartiality of the research reported.

## Funding

This work did not receive any specific grant from any funding agency in the public, commercial or not-for-profit sector.

## Author contribution statement

The two authors, D K and Y C, contributed to all aspects of the present paper (conception, experiments, data analyses, writing).
